# Supplementary material for: Endotoxemia Associated with Liver Disease Correlates with Systemic Inflammation and T Cell Exhaustion in Hepatitis C Virus Infection
Source: Cells. 2023 Aug 10;12(16):2034. doi: 10.3390/cells12162034 (PMC10453378; doi:10.3390/cells12162034)
Supplement: Supplementary file 1 [file cells-12-02034-s001.zip › cells-2483058-supplementary.pdf]

Figure S1.

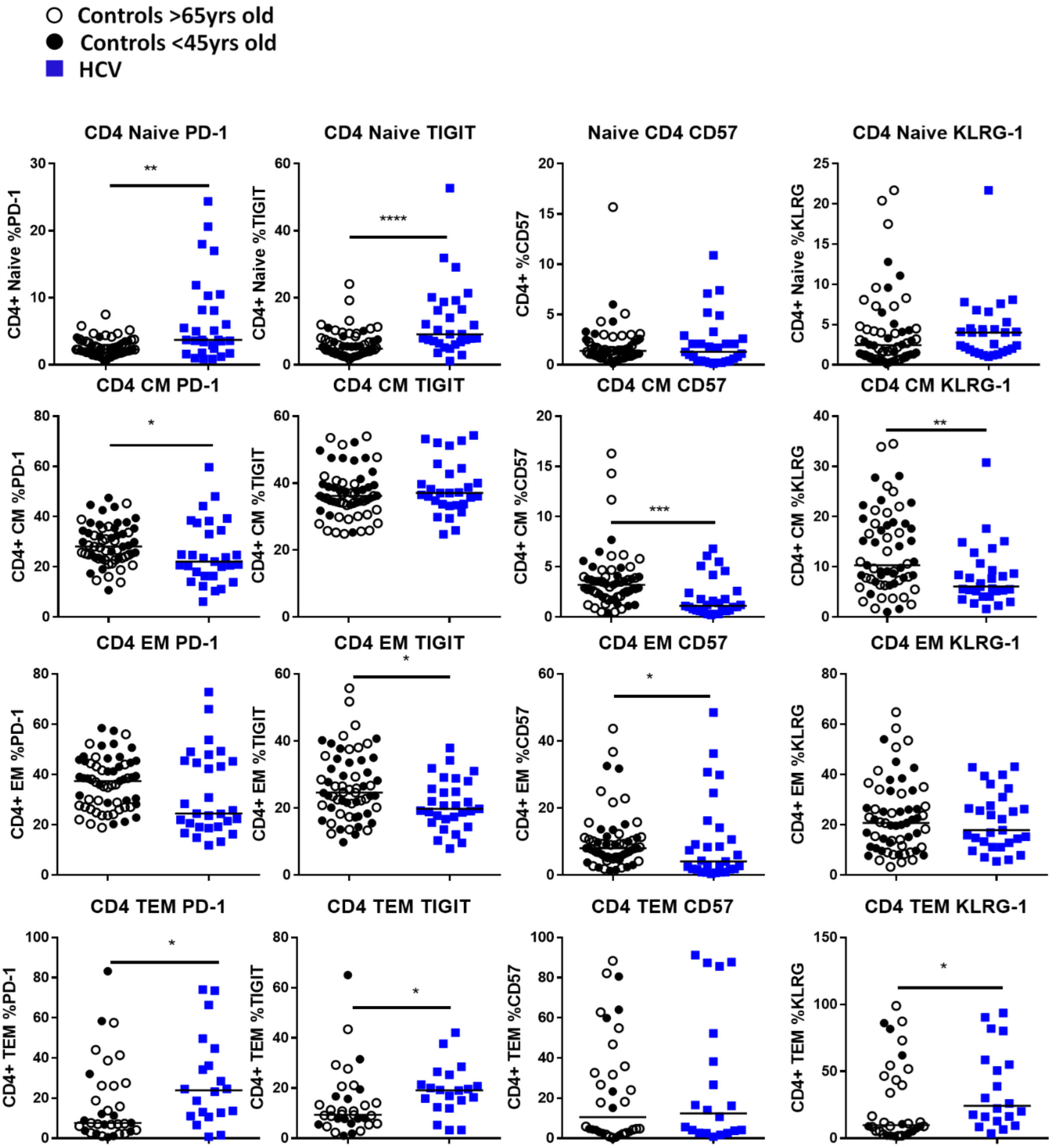

Controls >65yrs old
Controls <45yrs old
HCV
CD4 Naive PD-1
CD4 Naive TIGIT
Naive CD4 CD57
CD4 Naive KLRG-1
CD4+ Naive %PD-1
CD4+ Naive %TIGIT
CD4+ %CD57
CD4+ Naive %KLRG
CD4 CM PD-1
CD4 CM TIGIT
CD4 CM CD57
CD4 CM KLRG-1
CD4+ CM %PD-1
CD4+ CM %TIGIT
CD4+ CM %CD57
CD4+ CM %KLRG
CD4 EM PD-1
CD4 EM TIGIT
CD4 EM CD57
CD4 EM KLRG-1
CD4+ EM %PD-1
CD4+ EM %TIGIT
CD4+ EM %CD57
CD4+ EM %KLRG
CD4 TEM PD-1
CD4 TEM TIGIT
CD4 TEM CD57
CD4 TEM KLRG-1
CD4+ TEM %PD-1
CD4+ TEM %TIGIT
CD4+ TEM %CD57
CD4+ TEM %KLRG

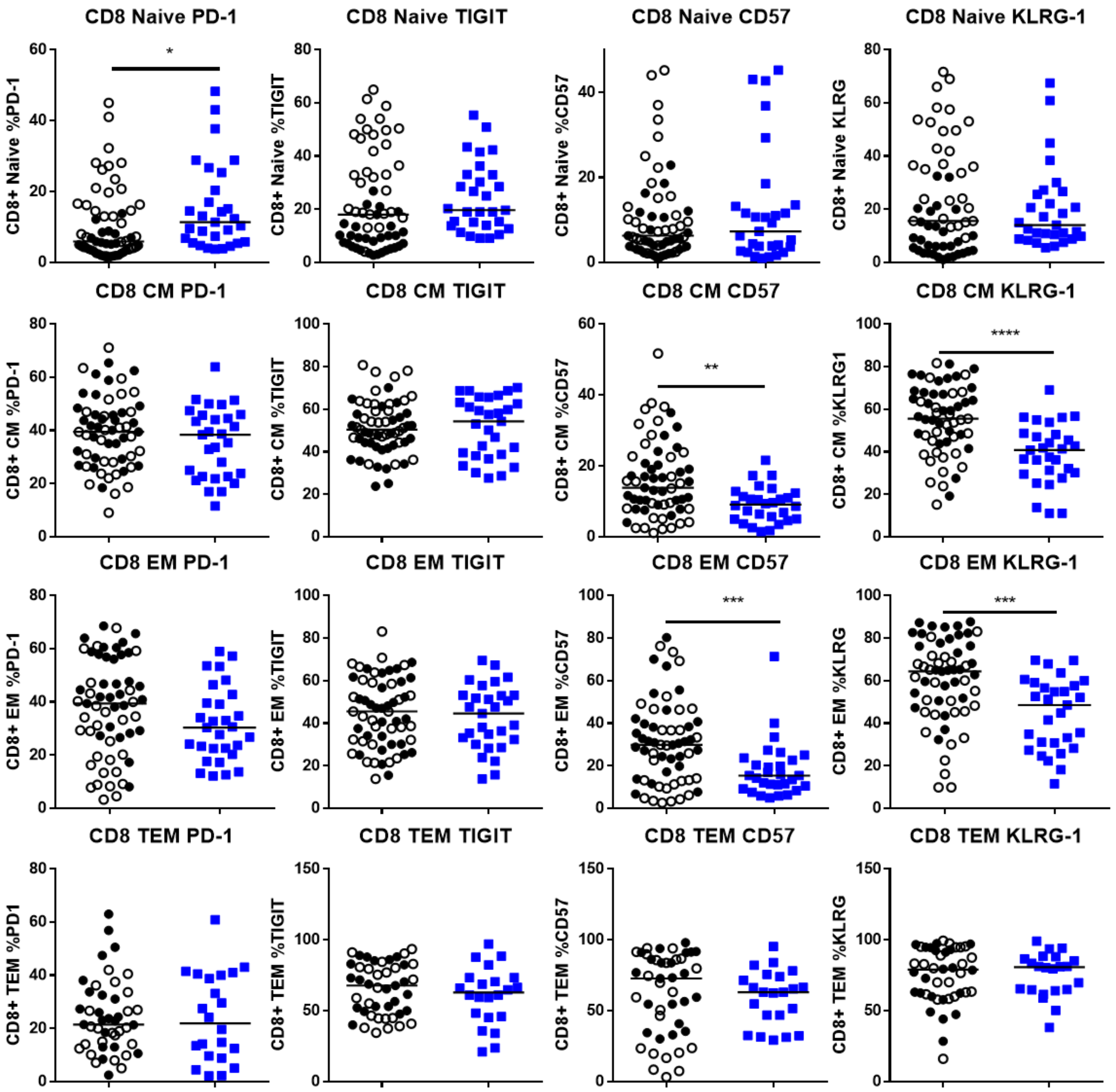

Figure S1. T cell exhaustion and senescence marker expression on T cell subsets
The proportion of T cell maturation subsets expressing markers of exhaustion (PD1, TIGIT) and senescence (CD57, KLRG1) were measured by flow cytometry. Proportions of live gated, CD3+ CD4+ or CD8+ naïve (CD45RA+ CD27+), central memory (CM) (CD45RA- CD27+), effector memory (EM) (CD45RA- CD27-), and terminal effector memory (TEM) (CD45RA+ CD27-) T cells expressing PD1, TIGIT, CD57, or KLRG1 are shown. Squares represent HCV-infected participants, closed circles represent uninfected controls <45 years old, and open circles represent uninfected controls >65 years old. Between groups comparisons made using Mann-Whitney U tests. P values < 0.05 considered statistically significant.

Figure S2a.

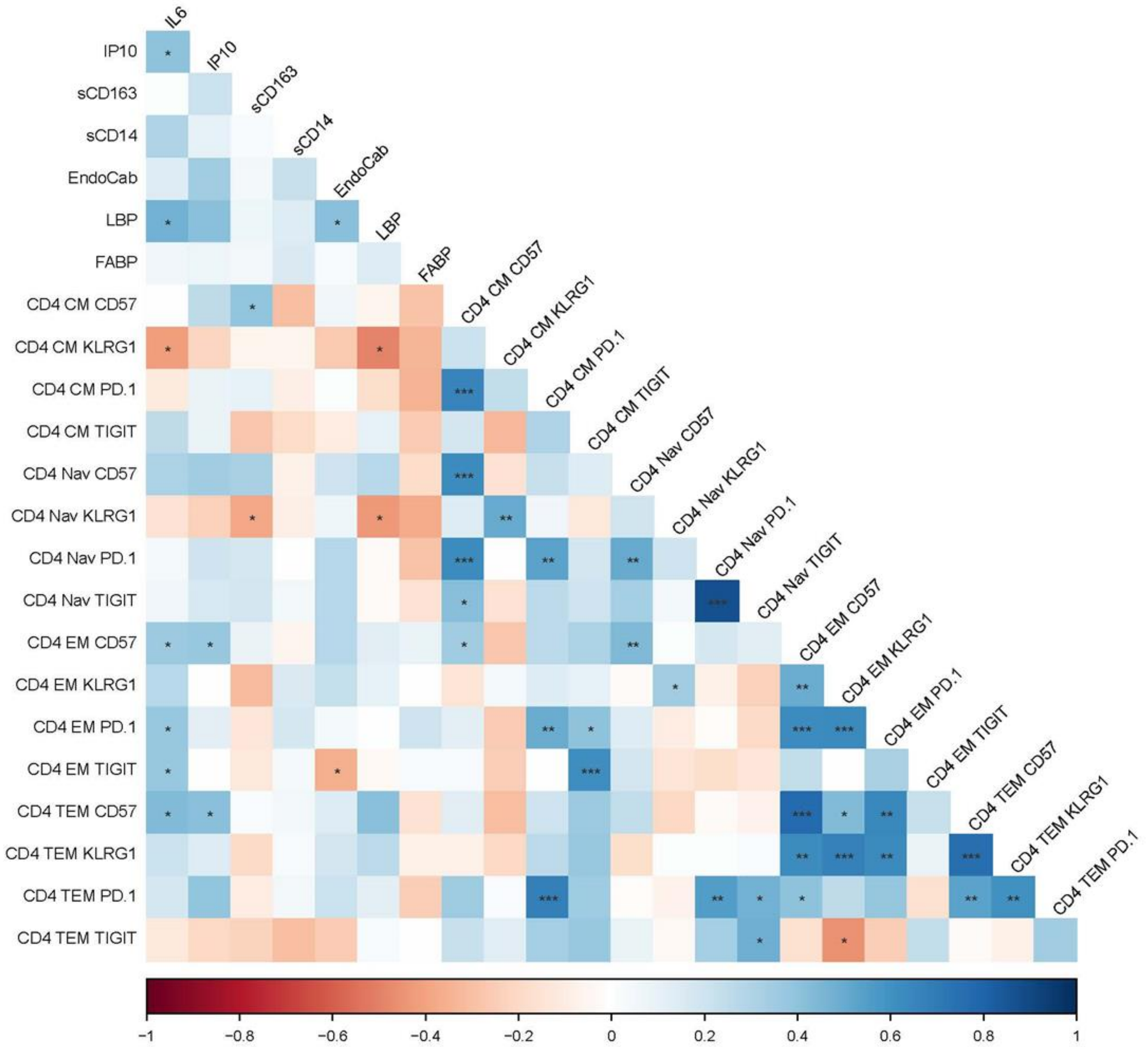
IL6
IP10
sCD163
sCD14
EndoCab
LBP
FABP
CD4 CM CD57
CD4 CM KLRG1
CD4 CM PD.1
CD4 CM TIGIT
CD4 Nav CD57
CD4 Nav KLRG1
CD4 Nav PD.1
CD4 Nav TIGIT
CD4 EM CD57
CD4 EM KLRG1
CD4 EM PD.1
CD4 EM TIGIT
CD4 TEM CD57
CD4 TEM KLRG1
CD4 TEM PD.1
CD4 TEM TIGIT
−1
−0.8
−0.6
−0.4
−0.2
0
0.2
0.4
0.6
0.8
1

Figure S2b.

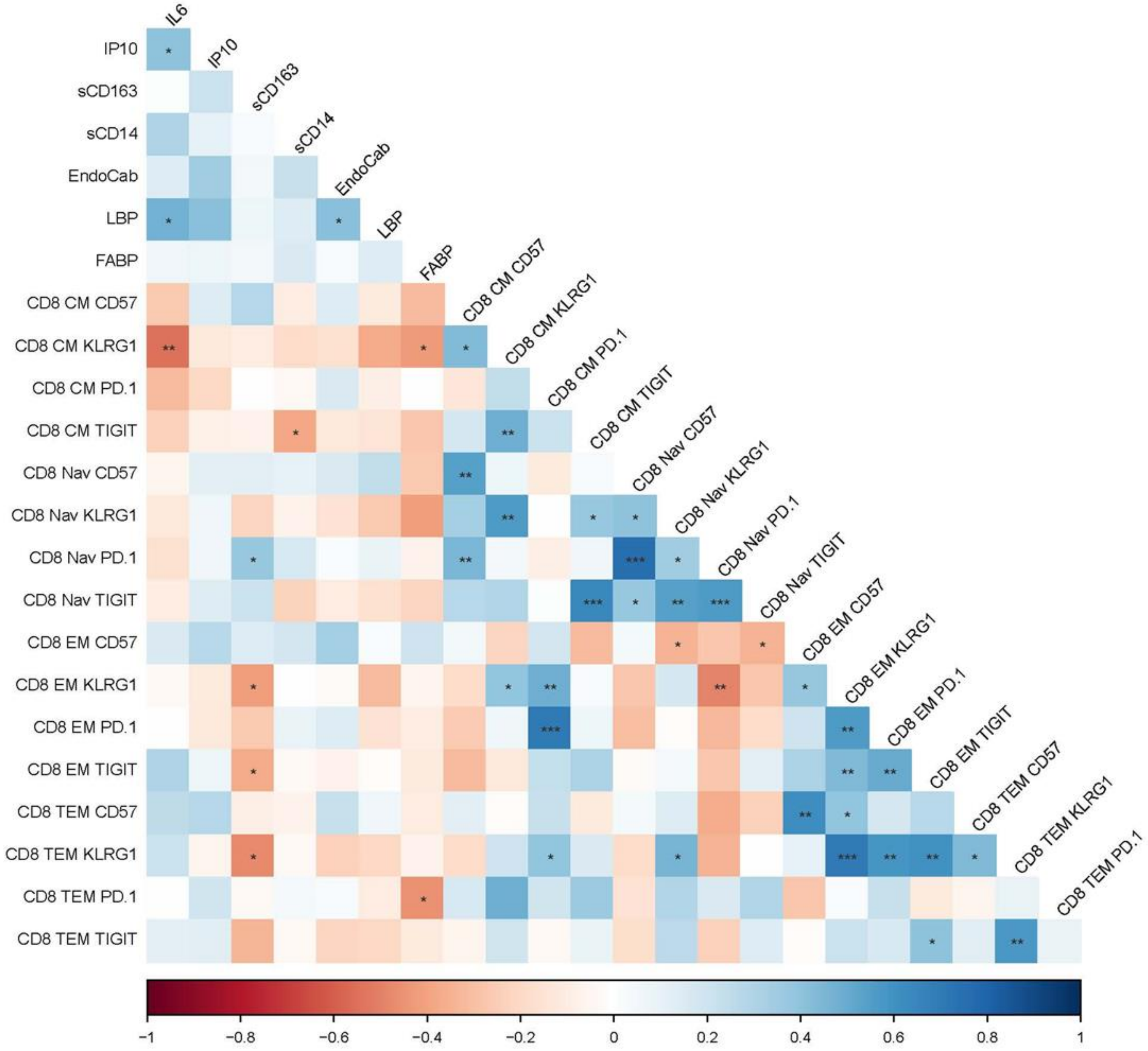

Figure S2. The association of T cell subset proportions of exhaustion/senescence with soluble indices of inflammation and endotoxemia

The proportions of CD4 (S2a) and CD8 (S2b) T cell maturation subsets expressing markers of exhaustion and senescence from HCV-infected participants were examined for correlations with levels of soluble markers of inflammation (IL-6, IP10, sCD163, sCD14) and endotoxemia (EndoCab, LBP, FABP) using Spearman's rank order statistical tests. The r value of correlation is represented by the color and color intensity. Positive correlations are blue, negative are red. Statistically significant correlations are represented with an asterisks, *p= <0.05; ** p= <0.001; ***p= <0.0001
